# CXCR4: From Signaling to Clinical Applications in Neuroendocrine Neoplasms

**DOI:** 10.3390/cancers16101799

**Published:** 2024-05-08

**Authors:** David Sanchis-Pascual, María Isabel Del Olmo-García, Stefan Prado-Wohlwend, Carlos Zac-Romero, Ángel Segura Huerta, Javier Hernández-Gil, Luis Martí-Bonmatí, Juan Francisco Merino-Torres

**Affiliations:** 1 Endocrinology and Nutrition Department, University and Politecnic Hospital La Fe (Valencia), 46026 Valencia, Spain; delolmo_margar@gva.es (M.I.D.O.-G.); merino_jfr@gva.es (J.F.M.-T.); 2 Joint Research Unit on Endocrinology, Nutrition and Clinical Dietetics, Health Research Institute La Fe, 46026 Valencia, Spain; 3 Nuclear Medicine Department, University and Politecnic Hospital La Fe (Valencia), 46026 Valencia, Spain; prado_ste@gva.es; 4 Patholoy Department, University and Politecnic Hospital La Fe (Valencia), 46026 Valencia, Spain; zac_carrom@gva.es; 5 Medical Oncology Department, University and Politecnic Hospital La Fe (Valencia), 46026 Valencia, Spain; segura_ang@gva.es; 6 Instituto de Tecnología Química, Universitat Politècnica de València, Consejo Superior de Investigaciones Científicas, 46022 Valencia, Spain; jahergi@upvnet.upv.es; 7 Medical Imaging Department, Biomedical Imaging Research Group, Health Research Institute, University and Politecnic Hospital La Fe, 46026 Valencia, Spain; luis.marti@uv.es; 8 Department of Medicine, University of Valencia, 46010 Valencia, Spain

**Keywords:** CXCR4, CXCL12, cancer, neuroendocrine neoplasm, radiotracer

## Abstract

**Simple Summary:**

Neuroendocrine neoplasms are a heterogeneous group of malignant tumors that originate from the diffuse endocrine system. They generally have a slow course and somatostatin receptor-targeted based management is the first line of treatment. However, high-grade tumors and neuroendocrine carcinomas have a poor prognosis and somatostatin receptor-targeted therapy is not effective. The membrane receptor CXCR4 has been studied in several neoplasms and it is known to be overexpressed in aggressive tumors and associated with a worse prognosis. However, there is a lack of evidence of its use in neuroendocrine neoplasms. For that reason, this review describes the significance of CXCR4 and its possible clinical applications in the diagnostic and therapeutic management of neuroendocrine neoplasms.

**Abstract:**

There are several well-described molecular mechanisms that influence cell growth and are related to the development of cancer. Chemokines constitute a fundamental element that is not only involved in local growth but also affects angiogenesis, tumor spread, and metastatic disease. Among them, the C-X-C motif chemokine ligand 12 (CXCL12) and its specific receptor the chemokine C-X-C motif receptor 4 (CXCR4) have been widely studied. The overexpression in cell membranes of CXCR4 has been shown to be associated with the development of different kinds of histological malignancies, such as adenocarcinomas, epidermoid carcinomas, mesenchymal tumors, or neuroendocrine neoplasms (NENs). The molecular synapsis between CXCL12 and CXCR4 leads to the interaction of G proteins and the activation of different intracellular signaling pathways in both gastroenteropancreatic (GEP) and bronchopulmonary (BP) NENs, conferring greater capacity for locoregional aggressiveness, the epithelial–mesenchymal transition (EMT), and the appearance of metastases. Therefore, it has been hypothesized as to how to design tools that target this receptor. The aim of this review is to focus on current knowledge of the relationship between CXCR4 and NENs, with a special emphasis on diagnostic and therapeutic molecular targets.

## 1. Introduction

### 1.1. Role of CXCL12 and CXCR4

Chemokines are a group of small molecules (~8–10 k Da) that belong to the cytokine family together with angiogenic factors, growth factors, or interferons and are secreted not only by neoplastic cells but also by macrophages, lymphocytes, or dendritic cells. Their main function is to stimulate chemotaxis of immune system cells as part of the inflammatory response through the interaction with fibroblasts or endothelial cells, while in neoplastic status they induce angiogenesis and sustain cell growth [1]. This action is exerted by binding the N-terminal domain, which is rich in the amino acid cysteine, to its specific receptor [2]. Depending on the distribution of this amino acid, four subtypes of cytokines are identified: CXC, CX3C, CC, and C [3].

Among the 50 types of chemokines known today, the chemokine CXCL12, which is also recognized as stromal cell-derived factor-1 (SDF-1) [4], has some characteristics that make it different from the rest of its family. Firstly, it is the only cytokine whose mRNA can be subjected to a process known as differential splicing, which is why up to six variants of this molecule have been recognized in humans (α to ϕ) [5,6]. Secondly, it is a chemokine with an almost exclusive affinity for a single receptor, with nothing to do with the promiscuity of the rest of the cytokines [7]. Until a few years ago, CXCR4 was recognized as the only natural receptor for CXCL12, although it has been discovered that it can also mediate its action through interaction with the atypical chemokine receptor type 3 (ACKR3), previously known as chemokine C-X-C motif receptor 7 (CXCR7) [8,9]. 

CXCL12 is probably the most important cytokine that binds to CXCR4 but this receptor does not only bind to this type of molecule. Different ligands for CXCR4 have been recognized, most notably macrophage inhibitory factor (MIF) [10] and ubiquitin [11,12].

### 1.2. Structure and Signaling Pathway of the CXCL12-CXCR4-ACKR3 Axis

CXCR4 is a molecular structure that has also presented different names throughout history. It was initially called leukocyte-derived seven-transmembrane receptor (LESTR) when it was isolated from a human blood monocyte cDNA library [13]. It has also been known as cluster of differentiation 184 (CD184) or fusin. The latter name refers to the ability of the human immunodeficiency virus 1 (HIV-1) to infect human cells by the process of fusion following the binding of its glycoprotein 120 (gp120) [14]. Although its natural ligand is the chemokine CXCL12 (as mentioned before), there is greater evidence that it has a wider spectrum of interactions with other molecules. In fact, it also recognizes ligands as small proteins like ubiquitin or the macrophage migration inhibiting factor (MIF) [15,16]. This receptor belongs to the family of G protein-coupled receptors (GPCRs), which are characterized by the presence of seven membrane-spanning α-helical segments separated by alternating intracellular and extracellular loop regions [17]. The intracytoplasmic domain of the receptor remains in contact with a heterotrimeric G protein that is composed of a G_α_, G_β_, and G_γ_ subunits and, when the interaction between CXCL12 and CXCR4 occurs, the exchange of guanosine diphosphate (GDP) for triphosphate (GTP) leads to a complex process in which a GTP-bound G_α_ monomer and a G_βγ_ dimer are released [18].

**Figure 1:**
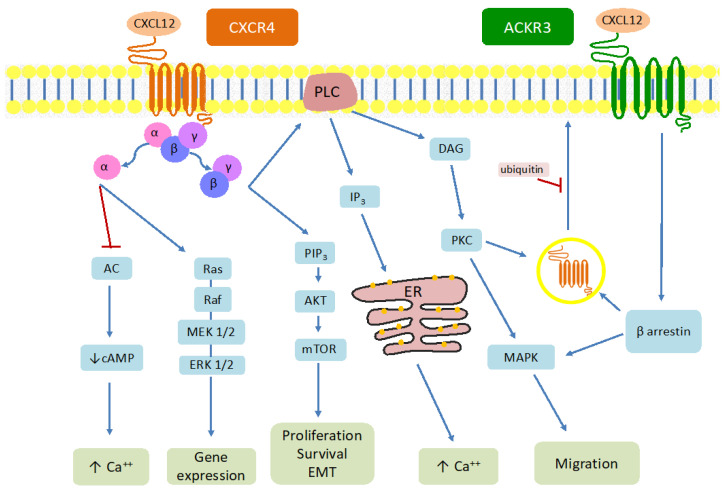
Representation of the signaling pathway in the activation of CXCR4 (left) and ACKR3 (right). Blue arrows mean activation while red arrows represent metabolic pathway inhibition. Note that G proteins and calcium do not participate as second messengers after binding CXCL12 to ACKR3. Acronyms: C-X-C motif chemokine ligand 12 (CXCL12), chemokine C-X-C motif receptor 4 (CXCR4), atypical cytokine receptor type 3 (ACKR3), protein kinase C (PKC), adenylate cyclase (AC), adenosine 3′,5′-cyclic monophosphate (cAMP), extracellular signal-regulated kinases (ERK), mitogen-activated protein kinase (MAPK), diacylglycerol (DAG), inositol-(1,4,5)-triphosphate (IP3), phosphatidylinositol triphosphate (PIP3), phospholipase C (PLC), mammalian target of rapamycin (mTOR), endoplasmic reticulum (ER).

The G_α_ subunit produces an inhibition of the adenylate cyclase leading to an increase in the intracellular calcium (Figure 1) mediated by the decrease in the concentration of adenosine 3′,5′-cyclic monophosphate (cAMP). It also interacts directly with the Src family of tyrosine kinases and then activates the signaling pathway of MEK1/2-Erk1/2 [19]. The G_βγ_ subunit activates phosphatidyl-inositol-3-OH kinase (PI3K) and consequently generates an increase in phosphatidylinositol triphosphate (PIP3), while the interaction with phospholipase C generates diacylglycerol (DAG) and inositol-(1,4,5)-triphosphate (IP3). IP3 increases intracellular calcium deposition after outflow from the endoplasmic reticulum (ER), while DAG interacts with protein kinase C and mitogen-activated protein kinase (MAPK) [20].

When CXCL12 binds ACKR3/CXCR7, a different signaling pathway is developed because of the biochemical difference between classical and atypical chemokine receptors, which basically boils down to the fact that atypical cytokine receptors (ACKRs) lack G proteins and its effects are calcium-independent [21]. The signal pathway through β-arrestin proteins becomes the main way the ACKR3 activation leads to its tumorigenic properties. β-arrestins increase the MEK/ERK axis and the protein kinase B (also known as Akt) activity [22]. The binding of CXCR4 to its agonist ligand results in phosphorylation and internalization of the receptor [23,24]. However, once inside the cell, it can be recycled and transported back to the plasma membrane or it can be degraded in the cell lysosomes [25]. The first scenario occurs in a PKC-mediated phenomenon [26], whereas the second case takes place after interaction with E3 ubiquitin ligase [27].

## 2. CXCR4 and Cancer

Firstly, the involvement of CXCR4 as a co-receptor in HIV infection overshadowed its potential as a tumorigenesis-related agent and it was not until 1999 when Burger et al. noticed that this protein favored migration of B cells in chronic lymphocytic leukemia. Since then, the link between CXCR4 and tumoral disease has been reviewed and, for instance, the implication of CXCR4 in more than 23 cancers is well known [28,29,30]. Considering that CXCR4 functions involve the promotion of cell growth, proinflammatory cell recruitment, angiogenesis, and cell migration, it is not surprising that the pathological activation of this receptor favors the development of tumoral disease. To be more accurate, the hyperactivation of the CXCL12/CXCR4/AKR3 axis is associated with increased tumor size, lower degree of cell differentiation, higher probability of recurrence, worse response to chemotherapy, and decreased overall survival [31,32]. The role it plays in cell growth and its different effects on stromal tissue have placed this receptor in the spotlight of the scientific community. CXCR4 has been studied in practically all the different types of cancer because its expression is independently associated with decreased survival [33]. In fact, it is being investigated as to whether it could be a pan-cancer marker of the microenvironment status [34].

The presence of metastases drastically worsens cancer prognosis and CXCR4 is closely related to this phenomenon in various solid tumors. It is hypothesized that the upward adjustment of the CXCR4/CXCL12 axis occurs in organs on which metastases frequently settle such as the liver, lung, brain, or bone [5,35,36] and this fact can be ratified if it is taken into account that the blockade of this axis leads to metastatic dissemination in animal studies [37,38]. Regarding the possible underlying mechanisms, the influence on the epithelial–mesenchymal transition (EMT) is postulated. This is a process characterized by the disarticulation of tight junctions and loss of apicobasal polarity [39] that facilitates distant dissemination and invasion of different organs by the acquisition of a mesenchymal phenotype. This process involves interleukin 11 [40], the NF-kB receptor [41], and CXCR4 [42,43].

Lastly, CXCR4 is closely related not only to solid tumors but also to the hematopoietic system [44]. Such is the case that the CXCR4/CXCL12-knockout mice exhibit specific characteristics which consist of heart malformations, abnormal cerebellar development, and absence of myelopoiesis and B lymphopoiesis [45,46,47]. This phenomenon can be explained if we take into consideration that CXCL12 is one of the most relevant cytokines involved in the chemotactic response of hematopoietic stem cells (HSCs) [48]. Having this ligand-receptor axis intact results necessary not only for the homing of HSCs through the bone marrow but also in retaining them in the hematopoietic microenvironment [49,50]. This knowledge has led to the development of strategies that target this level, such as the CXCR4 antagonist plerixafor, which is used in bone marrow transplant in patients with multiple myeloma or non-Hodgkin lymphoma due to its ability to mobilize HSCs from the bone stroma to the peripheral blood [51].

## 3. CXCR4 and NENs

### 3.1. Introduction to Neuroendocrine Neoplasms

NENs are a heterogeneous group of malignant tumors whose origin relies in the cells of the diffuse endocrine system, which are scattered throughout the human body, although the most frequent locations are in the gastrointestinal (GI) tract or in the lung. The incidence of NENs varies substantially according to the location of the primary tumor, being approximately 3.56 new cases per 100,000 in gastroenteropancreatic NENs (GEP-NENs), 1.49/100,000 in bronchopulmonary NENs (BP-NENs), and 0.84/100,000 in unknown primary NENs [52]. It is important to highlight the association of NENs with genetic syndromes such as multiple endocrine neoplasia syndrome type 1 [53]. NENs can be classified depending on whether they produce biologically active substances or not. Currently, it is considered that about 60% of NENs are non-functioning [54]. Carcinoid syndrome is the most common of the many syndromes that can develop due to hormone production [55] (such as insulinoma, glucagonoma, and gastrinoma).

The expression of somatostatin receptors (SSTR) on the cell membrane is a typical feature of NENs and it has diagnostic as well as therapeutic approaches [56]. In fact, the ability to diagnose NENs has improved substantially thanks to the incorporation of gallium-68(68Ga)-labeled DOTA tracers, such as DOTA-TOC, DOTA-TATE, and DOTA-NOC because of their sensitivity and specificity that reach 97% and 92%, respectively [57], compared to Indium-111 scintigraphy (sensitivity 72% and specificity 92%). 

Somatostatin analogs (SSA) constitute the first line treatment in NENs due to an antisecretory as well as an antiproliferative effect. In fact, administration of both octreotide [58] or lanreotide [59] has demonstrated increases in progression-free survival (PFS) versus placebo (14.3 vs. 6.0 months, HR 0.34, and >27 vs. 18 months, HR 0.47, respectively) in GEP-NENs. Regarding BP-NENs, only lanreotide has shown benefits in PFS [60] (16.6 vs. 13.6 months, HR 0.90). There are five types of SSTRs, although the drugs currently available focus on SSTR2A and SSTR5 agonism [61]. Lanreotide and octreotide mainly stimulate SSTR2 while pasireotide exerts its action after binding to SSTR2 and SSTR5. In general, NENs are indolent and slow-growing tumors. The main prognostic factor for GEP-NENs is the tumor grade according to the latest WHO classification, which takes into account cytologic features, the number of mitoses per field, and the Ki-67 proliferation index [62]. BP-NENs are governed by a similar classification but this does not take into account the proliferation index but the presence of necrosis on histology [63]. Four variants can be recognized: typical carcinoid (TC), atypical carcinoid (AC), large-cell neuroendocrine carcinoma (LCNC), and small-cell neuroendocrine carcinoma (SCNC).

### 3.2. Implications of CXCR4 Expression in NENs

The molecular study of NENs involves the detection and evaluation of multiple membrane targets, among which SSTRs are the most important. The more the SSTR is expressed (especially SSTR2A), the lower the grade and therefore the better the prognosis of NENs [64,65,66,67,68,69]. As with other types of neoplasms, the chemokine receptor CXCR4 is becoming increasingly relevant to researchers in the field of NENs. Circelli et al. demonstrated that the PI3K/Akt/mTOR pathway is enhanced both in GEP-NENs and in BP-NENs cell lines throughout an upregulation of the CXCR4-CXCL12 axis [70]. Indeed, the hyperactivation of this intracellular pathway has led to the development of mTOR inhibitors for the treatment of pancreatic NENs [71,72].

Among the multiple factors that determine the functioning of the CXCL12-CXCR4 axis, the hypoxia phenomenon plays a fundamental role in carcinogenesis [73,74,75] and in the homeostasis of these molecules [76,77]. The hypoxia-inducible factors 1α and 2α (HIF1α and HIF2α) increase the expression of CXCR4, confer greater aggressiveness, and result in lower survival in patients with ileal NENs [78,79]. Kaemmerer et al. demonstrated an inverse relationship between CXCR4 expression and overall survival (OS) in GEP-NENs, since those patients with a marked expression of this receptor had a lower, although not significant, OS compared to those with a weak expression (34.0 vs. 50.0 months, *p* = 0.068). This expression was higher in high-grade tumors (differentiating also between grades 3a and 3b) versus low-grade tumors. Furthermore, a positive correlation was identified between CXCR4 and Ki-67 index (r 0.39; *p* < 0.001) and with SSTR5 expression (r 0.27; *p* = 0.003), while SSTR2A expression showed a robust inverse correlation (r −0.50; *p* < 0.001) [80]. 

No clear agreement exists about the relationship between CXCR4 expression and GEP-NEN location. While Mai et al. showed higher expression in those whose primary tumor is located in the appendix or colon (*p* = 0.024) [81], Popa et al. showed no statistical differences among them, but greater expression in colonic primary tumors but less immunoreactive intensity in appendix ones [82]. Interestingly, no statistical differences were found in both studies between primary tumors and metastases in the intensity of expression. In relation to hormone production, it appears that expression is higher in those non-functioning neoplasms (*p* = 0.019) [81]. Regarding BP-NENs, an inverse correlation with OS has also been shown. TC and AT tend to show lower expression of CXCR4 but are high and intense in SCLC [83]. The role that CXCR4 plays in the EMT is crucial in the pathogenesis of metastatic disease in both GEP and BP-NENs [84,85,86,87]. It seems that among multiple target organs, bone involvement is intimately influenced by the overexpression of this receptor, in both in vitro [88] and in vivo [89] studies. 

### 3.3. CXCR4 as a Target for Imaging Diagnosis on NENs

The use of computed tomography (CT) and magnetic resonance imaging (MRI) scans is essential in the diagnosis and staging of tumoral disease. However, the use of functional imaging techniques through the administration of radiotracers has become a cornerstone in the management of patients with NENs. The main molecular targets in the study of NENs are somatostatin receptors, especially SSTR2 and SSTR5. The first imaging techniques that emerged were 111In-DTPA-Pentetreotide (Octreoscan^®^) and 99mTC-EDDA-HYNIC-Thr3-octreotide (Tektrotyd^®^) scintigraphy with improved spatial resolution using single photon emission tomography (SPECT/CT). Diagnostic performance was subsequently increased with the introduction of radiopharmaceuticals suitable for positron emission tomography (PET/CT) such as 68Ga-labeled tracers. However, the expression of SSTRs decreases with the increasing tumor grade, which influences the 68Ga-PET/CT sensitivity (72.2% in G1 vs. 40.8% in G3 NENs) and maximum standardized uptake value (SUVmax) (29.2 in G1 vs. 12.8 in G3 NENs) [90], so SSTR targeting may be less useful in the diagnosis and follow-up of dedifferentiated NENs. In this scenario, ^18^F-fluorodeoxyglucose (18F-FDG) PET/CT provides additional information and insight into the metabolic state of the neoplastic lesions [91,92]. However, this technique is not free of interferences that may hinder its correct interpretation [93] and it is therefore necessary to investigate alternative molecular targets for lesions with a lower degree of differentiation.

In 2008, Uy et al. developed the drug plerixafor (AMD3100), a CXCR4 antagonist that prevents binding of its ligand CXCL12/SDF-1 and is used for stem cell mobilization from bone marrow prior to hematopoietic progenitor transplantation [94]. In 2014, Aghanejad et al. developed a 68Gallium-plerixafor radiotracer that demonstrated its potential utility in the field of Oncology by detecting breast cancer cells in an in vivo mouse model [95]. However, previously, Gourni et al. designed a molecule composed of a cyclic peptide CPCR4-2 labeled with 68Ga (cyclo(D-Tyr1-[NMe]-D-Orn2-[4-(aminomethyl) benzoic acid,68Ga-DOTA]-Arg3-2-Nal4-Gly5, also known as pentixafor), which has revealed higher specificity for CXR4 and greater in vivo stability for the study of malignant neoplasms in humans [96,97,98]. 68Ga-Pentixafor seems to be an interesting future tool and therefore studies are being carried out to show the usefulness of this in various types of neoplasms [99].

Regarding NENs, Werner et al. were the first to noninvasively evaluate CXCR4 expression by 68Ga-Pentixafor PET/CT compared with 68Ga-DOTA-TOC and 18F-FDG PET/CT in 12 patients with GEP-NENs. 68Ga-Pentixafor was negative in all G1-NENs while 68Ga-DOTA-TOC and 18F-FDG PET/CT identified lesions in 12/12 and 11/12 patients, respectively. In G2-NENs, 68Ga-Pentixafor was positive in half of the cases (2/4) whereas both 68Ga-DOTA-TOC and 18F-FDG were positive in all of them and in G3-NENs both 68Ga-Pentixafor and 68Ga-DOTA-TOC confirmed positivity in four out of five patients when 18F-FDG was positive in five out of five of the cases. These data agree with what has been published to date on the lower expression of SSTR and the increase in CXCR4 at higher tumor grade. However, the results further support the use of 18F-FDG against direct targeting of CXCR4 with pentixafor. In addition, the number of lesions identified was markedly lower compared to the other radiotracers, both overall (69 lesions for 68Ga-Pentixafor, 127 for 18F-FDG, and 245 for 68Ga-DOTA-TOC) and stratified by WHO tumor grading [100]. Interestingly, not only are there differences in the ability to detect lesions in different patients but intraindividual variability has also been shown in which some G3-NENs lesions may be positive for 18F-FDG and negative for 68Ga-Pentixafor or vice versa. That makes the management of patients with NENs more complex because of the existence of multiple lesions with different molecular behaviors [101]. 

To evaluate the diagnostic potential of CXCR4 labeling in dedifferentiated tumors, Weich et al. confronted 18F-FDG and 68Ga-Pentixafor PET/CT in 11 patients newly diagnosed from GEP-NEC and studied IHC expression of CXCR4. In a per-patient analysis, 18F-FDG-avid lesions were detected in all patients while 68Ga-Pentixafor was positive in 10/11 patients. In a per lesion analysis, the ability of 18F-FDG to reveal more lesions in comparison with 68Ga-Pentixafor was shown (102 vs. 42 lesions, *p* < 0.001). In relation to radiotracer uptake intensity, 18F-FDG showed a higher SUVmax in contrast to 68Ga-Pentixafor (12.8 ± 9.8 vs. 5.2 ± 3.7, *p* < 0.001) and greater tumor-to-background ratios (TBR) (7.2 ± 7.9 vs. 3.4 ± 3.0, *p* < 0.001). With respect to IHC, the overall CXCR4 expression was cataloged as low in 7/11 patients and there was no correlation between the intensity of CXCR4 expression and the 68Ga-Pentixafor uptake in biopsies [102].

With regard to BP-NENs, the correlation between the 68Ga-Pentixafor uptake in PET/CT images and the CXCR4 expression by mean fluorescence index and IHC was studied. Although there was an increased uptake in all patients, no correlation was found to both cytologic features [103]. However, a study comparing the usefulness of 68Ga-Pentixafor versus other types of radiotracers in BP-NENs has not yet been developed. In addition, 68Ga-Pentixafor has not shown an association with clinical parameters such as OS or PFS both in GP-NENs and SCLC, although it does appear to be related to leukocyte and platelet counts [104].

Among the multiple interrelated processes in CXCR4 homeostasis, the Wnt/β-catenin molecular pathway is fundamental in the correct functioning of the CXCL12-CXCR4 axis [105,106] and is also deregulated in about 25% of patients with GEP, lung, or thymus NENs [107]. For this reason, the possibility of modulating the expression density of CXCR4 and its 68Ga-Pentixafor binding capacity in NEN cell lines has been studied, achieving promising results that open the door to future studies with Wnt inhibitors or activators [108].

### 3.4. CXCR4 Targeting as Treatment of NENs

**Figure 2:**
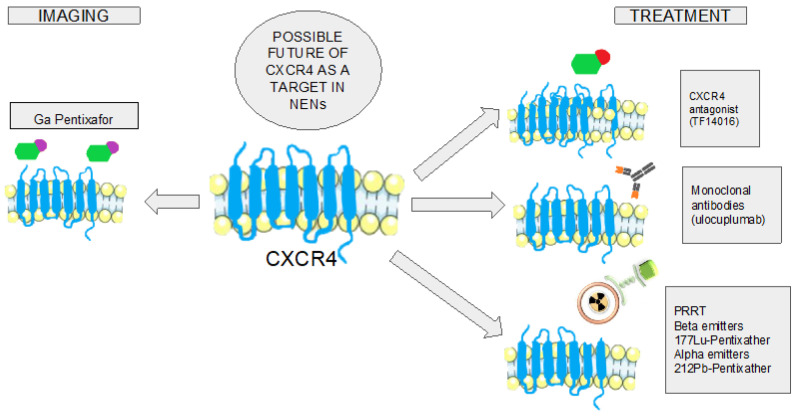
Schematic representation that summarizes the future of CXCR4 as a target in NENs, in both diagnostic and therapeutic approaches. Acronyms: peptide receptor radionuclide therapy (PRRT), Gallium (Ga), Lead (Pb), and Lutetium (Lu).

Precision medicine consists of individualizing treatment according to the specific characteristics of each patient and neoplasm. In the case of NENs, a good example is treatment with SSA, which performs its action specifically against neoplastic cells that express SSTR in their plasma membrane. However, sometimes, this treatment is not sufficient and it is necessary to identify new therapeutic targets. CXCR4 emerges as a possible target and the selective approach against it can be carried out using different therapeutic strategies [109] (Figure 2).

#### 3.4.1. Synthetic Peptides

Administration of TF14016, a direct CXCR4 inhibitor, has been shown in animal models to decrease the number and size of pulmonary metastases in SCLC. In addition, a lower expression of vascular endothelial cell growth factor was recorded [110]. A cyclic peptide antagonist called LY2510924 was studied in a phase II trial in patients with SCLC added to carboplatin/etoposide but did not show efficacy (PFS 5.88 vs. 5.85, *p* = 0.9806) although its toxicity profile was acceptable [111]. Although there is limited information about the treatment of NENs with this type of molecules, there are peptides such as balixafortide, motixafortide, and mavorixafor that have been studied in the treatment of solid neoplasms [112,113,114], HSC mobilization prior to bone marrow transplantation [115,116], and even in rare warts, hypogammaglobulinemia, immunodeficiency, and myelokathexis (WHIM) syndrome [117] but not yet neither in GEP nor BP-NENs.

#### 3.4.2. Monoclonal Antibodies

Several antibodies against CXCR4 have been studied, although most trials are in the early stages and evidence in NENs is limited [118]. For the time being, no in vivo studies have been developed and the information available comes from in vitro studies. The effect of ulocuplumab (a monoclonal antibody that prevents CXCL12 binding) has been studied in pancreatic NENs [119]. Although it has not been shown to exert a cytolytic effect on tumor cells, a reduced migration toward the liver and bone by inhibiting EMT has been observed. Intriguingly, Yingnan Si et al. developed dual SSTR2/CXCR4 targeted extracellular vesicles-delivered combined therapy through monoclonal antibodies against pancreatic, thyroid, and lung NENs [120]. This experimental treatment showed an anticancer efficacy both in vitro and in vivo models and no systemic toxicity was reported. 

#### 3.4.3. Peptide Receptor Radionuclide Therapy

Theragnosis is a medical approach combining diagnosis and therapy to tailor treatment strategies for individual patients, primarily used in cancer care to identify specific receptors and then target them with precise radiotracer. 

As mentioned above, plerixafor is a CXCR4 antagonist mainly used in hematopoietic stem cell transplants. However, it has also been studied for stem cell collection in patients with a NEN massive bone marrow infiltration, prior to the administration of 177Lu-DOTATATE, and initiated after the failure of a granulocyte-colony stimulating factor [121]. NENs are a type of tumor in which peptide receptor radionuclide therapy (PRRT) has been implemented since the publication of the trial NETTER-1 in 2017 [122]. This trial showed the superiority of 177Lu-DOTATATE versus SSA high-dose monotherapy in terms of PFS. Although a non-significant improvement in OS was subsequently identified (36.3 months vs. 40 months in the PRRT-Lu arm, *p* = 0.30), this effect was attributed to the high rate (36%) of cross-over of patients in the control arm to PRRT after progression [123].

The role of 68Ga-Pentixafor in the diagnosis of high-grade NENs and dedifferentiated NECs has been investigated. However, due to its altered affinity for CXCR4 when interacting with metal-chelate conjugates and its relatively fast clearance [124], 68Ga-Pentixafor does not appear to be a valid tool for the therapeutic management of malignancies. Thus, Schottelius et al. designed a novel molecule with improved pharmacokinetics called pentixather, which was labeled with 177Lu [125]. Most of the available evidence for this novel radiopharmaceutical comes from its endoradiotherapeutic use in hematologic malignancies. It has been shown to elicit high responses and decrease 18F-FDG uptake in multiple myeloma lesions both bound to 177Lu and 90Y [126,127]. It has also demonstrated utility in refractory acute leukemia and diffuse large-cell lymphoma [128,129] and may be useful in glioblastoma cells [130]. The available evidence for pentixather in the treatment of NENs comes only from BP-NENs in animal studies. On the one hand, 177Lu-Pentixather has been shown to decrease tumor growth and increase OS in mice with SCLC [131]. On the other hand, the administration of 212Pb-Pentixather associated with a thioredoxin reductase inhibitor caused a delay in tumor growth in mice with SCLC xenograft [132].

## 4. Conclusions

CXCR4 and its ligand CXCL12 are essential in the tumorigenesis and development of NENs. It appears that SSTRs and CXCR4 maintain an antagonistic relationship that favors the latter in high-grade, dedifferentiated, and metastatic tumors. Consequently, current research is focusing on selectively targeting this membrane receptor. At the moment, it seems that targeted diagnosis using ^68^Ga-Pentixafor does not provide more information than ^18^F-FDG although there are mechanisms that may influence its uptake and be relevant in the future. The treatment of NENs with molecules specifically directed against CXCR4 is in the preclinical phase, although the data on radiopharmaceuticals such as ^177^Lu-Pentixather or ^212^Pb-Pentixather in the theragnosis treatment of BP-NENs are encouraging. 

## Data Availability

Data are contained within the article.

## References

[1] Raman D., Baugher P.J., Thu Y.M., Richmond A. (2007). Role of Chemokines in Tumor Growth. Cancer Lett..

[2] Le Y., Zhou Y., Iribarren P., Wang J. (2004). Chemokines and Chemokine Receptors: Their Manifold Roles in Homeostasis and Disease. Cell. Mol. Immunol..

[3] Bachelerie F., Ben-Baruch A., Burkhardt A.M., Combadiere C., Farber J.M., Graham G.J., Horuk R., Sparre-Ulrich A.H., Locati M., Luster A.D. (2014). International Union of Pharmacology. LXXXIX. Update on the Extended Family of Chemokine Receptors and Introducing a New Nomenclature for Atypical Chemokine Receptors. Pharmacol. Rev..

[4] Bleul C.C., Fuhlbrigge R.C., Casasnovas J.M., Aiuti A., Springer T.A. (1996). A Highly Efficacious Lymphocyte Chemoattractant, Stromal Cell-Derived Factor 1 (SDF-1). J. Exp. Med..

[5] Yu L., Cecil J., Peng S.-B., Schrementi J., Kovacevic S., Paul D., Su E.W., Wang J. (2006). Identification and Expression of Novel Isoforms of Human Stromal Cell-Derived Factor 1. Gene.

[6] Righetti A., Giulietti M., Šabanović B., Occhipinti G., Principato G., Piva F. (2019). CXCL12 and Its Isoforms: Different Roles in Pancreatic Cancer?. J. Oncol..

[7] Griffith J.W., Sokol C.L., Luster A.D. (2014). Chemokines and Chemokine Receptors: Positioning Cells for Host Defense and Immunity. Annu. Rev. Immunol..

[8] Balabanian K., Lagane B., Infantino S., Chow K.Y.C., Harriague J., Moepps B., Arenzana-Seisdedos F., Thelen M., Bachelerie F. (2005). The Chemokine SDF-1/CXCL12 Binds to and Signals through the Orphan Receptor RDC1 in T Lymphocytes. J. Biol. Chem..

[9] Burns J.M., Summers B.C., Wang Y., Melikian A., Berahovich R., Miao Z., Penfold M.E.T., Sunshine M.J., Littman D.R., Kuo C.J. (2006). A Novel Chemokine Receptor for SDF-1 and I-TAC Involved in Cell Survival, Cell Adhesion, and Tumor Development. J. Exp. Med..

[10] Rajasekaran D., Gröning S., Schmitz C., Zierow S., Drucker N., Bakou M., Kohl K., Mertens A., Lue H., Weber C. (2016). Macrophage Migration Inhibitory Factor-CXCR4 Receptor Interactions. J. Biol. Chem..

[11] Tripathi A., Saini V., Marchese A., Volkman B.F., Tang W.-J., Majetschak M. (2013). Modulation of the CXC Chemokine Receptor 4 Agonist Activity of Ubiquitin through C-Terminal Protein Modification. Biochemistry.

[12] Scofield S.L.C., Daniels C.R., Dalal S., Millard J.A., Singh M., Singh K. (2018). Extracellular Ubiquitin Modulates Cardiac Fibroblast Phenotype and Function via Its Interaction with CXCR4. Life Sci..

[13] Loetscher M., Geiser T., O’Reilly T., Zwahlen R., Baggiolini M., Moser B. (1994). Cloning of a Human Seven-Transmembrane Domain Receptor, LESTR, That Is Highly Expressed in Leukocytes. J. Biol. Chem..

[14] Crump M.P., Gong J.H., Loetscher P., Rajarathnam K., Amara A., Arenzana-Seisdedos F., Virelizier J.L., Baggiolini M., Sykes B.D., Clark-Lewis I. (1997). Solution Structure and Basis for Functional Activity of Stromal Cell-Derived Factor-1; Dissociation of CXCR4 Activation from Binding and Inhibition of HIV-1. EMBO J..

[15] Bernhagen J., Krohn R., Lue H., Gregory J.L., Zernecke A., Koenen R.R., Dewor M., Georgiev I., Schober A., Leng L. (2007). MIF Is a Noncognate Ligand of CXC Chemokine Receptors in Inflammatory and Atherogenic Cell Recruitment. Nat. Med..

[16] Saini V., Staren D.M., Ziarek J.J., Nashaat Z.N., Campbell E.M., Volkman B.F., Marchese A., Majetschak M. (2011). The CXC Chemokine Receptor 4 Ligands Ubiquitin and Stromal Cell-Derived Factor-1α Function through Distinct Receptor Interactions. J. Biol. Chem..

[17] Rosenbaum D.M., Rasmussen S.G.F., Kobilka B.K. (2009). The Structure and Function of G-Protein-Coupled Receptors. Nature.

[18] Goldsmith Z.G., Dhanasekaran D.N. (2007). G Protein Regulation of MAPK Networks. Oncogene.

[19] New D.C., Wu K., Kwok A.W.S., Wong Y.H. (2007). G Protein-Coupled Receptor-Induced Akt Activity in Cellular Proliferation and Apoptosis. FEBS J..

[20] Mellado M., Rodríguez-Frade J.M., Mañes S., Martínez-A C. (2001). Chemokine Signaling and Functional Responses: The Role of Receptor Dimerization and TK Pathway Activation. Annu. Rev. Immunol..

[21] Thelen M., Thelen S. (2008). CXCR7, CXCR4 and CXCL12: An Eccentric Trio?. J. Neuroimmunol..

[22] Rajagopal S., Kim J., Ahn S., Craig S., Lam C.M., Gerard N.P., Gerard C., Lefkowitz R.J. (2010). β-Arrestin- but Not G Protein-Mediated Signaling by the “Decoy” Receptor CXCR7. Proc. Natl. Acad. Sci. USA.

[23] Haribabu B., Richardson R.M., Fisher I., Sozzani S., Peiper S.C., Horuk R., Ali H., Snyderman R. (1997). Regulation of Human Chemokine Receptors CXCR4: Role Of Phosphorylation in Desensitization and Internalization. J. Biol. Chem..

[24] Signoret N., Rosenkilde M.M., Klasse P.J., Schwartz T.W., Malim M.H., Hoxie J.A., Marsh M. (1998). Differential Regulation of CXCR4 and CCR5 Endocytosis. J. Cell Sci..

[25] Marchese A., Chen C., Kim Y.-M., Benovic J.L. (2003). The Ins and Outs of G Protein-Coupled Receptor Trafficking. Trends Biochem. Sci..

[26] Signoret N., Oldridge J., Pelchen-Matthews A., Klasse P.J., Tran T., Brass L.F., Rosenkilde M.M., Schwartz T.W., Holmes W., Dallas W. (1997). Phorbol Esters and SDF-1 Induce Rapid Endocytosis and Down Modulation of the Chemokine Receptor CXCR4. J. Cell Biol..

[27] Marchese A., Raiborg C., Santini F., Keen J.H., Stenmark H., Benovic J.L. (2003). The E3 Ubiquitin Ligase AIP4 Mediates Ubiquitination and Sorting of the G Protein-Coupled Receptor CXCR4. Dev. Cell.

[28] Burger J.A., Burger M., Kipps T.J. (1999). Chronic Lymphocytic Leukemia B Cells Express Functional CXCR4 Chemokine Receptors That Mediate Spontaneous Migration beneath Bone Marrow Stromal Cells. Blood.

[29] Balkwill F. (2004). Cancer and the Chemokine Network. Nat. Rev. Cancer.

[30] Drury L.J., Ziarek J.J., Gravel S., Veldkamp C.T., Takekoshi T., Hwang S.T., Heveker N., Volkman B.F., Dwinell M.B. (2011). Monomeric and Dimeric CXCL12 Inhibit Metastasis through Distinct CXCR4 Interactions and Signaling Pathways. Proc. Natl. Acad. Sci. USA.

[31] Kim J., Takeuchi H., Lam S.T., Turner R.R., Wang H.-J., Kuo C., Foshag L., Bilchik A.J., Hoon D.S.B. (2005). Chemokine Receptor CXCR4 Expression in Colorectal Cancer Patients Increases the Risk for Recurrence and for Poor Survival. J. Clin. Oncol. Off. J. Am. Soc. Clin. Oncol..

[32] Scala S., Ottaiano A., Ascierto P.A., Cavalli M., Simeone E., Giuliano P., Napolitano M., Franco R., Botti G., Castello G. (2005). Expression of CXCR4 Predicts Poor Prognosis in Patients with Malignant Melanoma. Clin. Cancer Res. Off. J. Am. Assoc. Cancer Res..

[33] Zhao H., Guo L., Zhao H., Zhao J., Weng H., Zhao B. (2014). CXCR4 Over-Expression and Survival in Cancer: A System Review and Meta-Analysis. Oncotarget.

[34] Marquardt A., Hartrampf P., Kollmannsberger P., Solimando A.G., Meierjohann S., Kübler H., Bargou R., Schilling B., Serfling S.E., Buck A. (2023). Predicting Microenvironment in CXCR4- and FAP-Positive Solid Tumors—A Pan-Cancer Machine Learning Workflow for Theranostic Target Structures. Cancers.

[35] Janowski M. (2009). Functional Diversity of SDF-1 Splicing Variants. Cell Adhes. Migr..

[36] Müller A., Homey B., Soto H., Ge N., Catron D., Buchanan M.E., McClanahan T., Murphy E., Yuan W., Wagner S.N. (2001). Involvement of Chemokine Receptors in Breast Cancer Metastasis. Nature.

[37] Chow M.T., Luster A.D. (2014). Chemokines in Cancer. Cancer Immunol. Res..

[38] Darash-Yahana M., Pikarsky E., Abramovitch R., Zeira E., Pal B., Karplus R., Beider K., Avniel S., Kasem S., Galun E. (2004). Role of High Expression Levels of CXCR4 in Tumor Growth, Vascularization, and Metastasis. FASEB J. Off. Publ. Fed. Am. Soc. Exp. Biol..

[39] Thiery J.P. (2002). Epithelial-Mesenchymal Transitions in Tumour Progression. Nat. Rev. Cancer.

[40] Bockhorn J., Dalton R., Nwachukwu C., Huang S., Prat A., Yee K., Chang Y.-F., Huo D., Wen Y., Swanson K.E. (2013). MicroRNA-30c Inhibits Human Breast Tumour Chemotherapy Resistance by Regulating TWF1 and IL-11. Nat. Commun..

[41] Chu C.-Y.G., Chung L.W.K. (2014). RANK-Mediated Signaling Network and Cancer Metastasis. Cancer Metastasis Rev..

[42] Zhu Y., Yang P., Wang Q., Hu J., Xue J., Li G., Zhang G., Li X., Li W., Zhou C. (2013). The Effect of CXCR4 Silencing on Epithelial-Mesenchymal Transition Related Genes in Glioma U87 Cells. Anat. Rec. Hoboken NJ 2007.

[43] Cives M., Rizzo F., Simone V., Bisceglia F., Stucci S., Seeber A., Spizzo G., Montrone T., Resta L., Silvestris F. (2016). Reviewing the Osteotropism in Neuroendocrine Tumors: The Role of Epithelial-Mesenchymal Transition. Neuroendocrinology.

[44] Mehrpouri M. (2022). The Contributory Roles of the CXCL12/CXCR4/CXCR7 Axis in Normal and Malignant Hematopoiesis: A Possible Therapeutic Target in Hematologic Malignancies. Eur. J. Pharmacol..

[45] Nagasawa T., Hirota S., Tachibana K., Takakura N., Nishikawa S., Kitamura Y., Yoshida N., Kikutani H., Kishimoto T. (1996). Defects of B-Cell Lymphopoiesis and Bone-Marrow Myelopoiesis in Mice Lacking the CXC Chemokine PBSF/SDF-1. Nature.

[46] Ma Q., Jones D., Borghesani P.R., Segal R.A., Nagasawa T., Kishimoto T., Bronson R.T., Springer T.A. (1998). Impaired B-Lymphopoiesis, Myelopoiesis, and Derailed Cerebellar Neuron Migration in CXCR4- and SDF-1-Deficient Mice. Proc. Natl. Acad. Sci. USA.

[47] Zou Y.R., Kottmann A.H., Kuroda M., Taniuchi I., Littman D.R. (1998). Function of the Chemokine Receptor CXCR4 in Haematopoiesis and in Cerebellar Development. Nature.

[48] Wright D.E., Bowman E.P., Wagers A.J., Butcher E.C., Weissman I.L. (2002). Hematopoietic Stem Cells Are Uniquely Selective in Their Migratory Response to Chemokines. J. Exp. Med..

[49] Aiuti A., Webb I.J., Bleul C., Springer T., Gutierrez-Ramos J.C. (1997). The Chemokine SDF-1 Is a Chemoattractant for Human CD34+ Hematopoietic Progenitor Cells and Provides a New Mechanism to Explain the Mobilization of CD34+ Progenitors to Peripheral Blood. J. Exp. Med..

[50] Peled A., Grabovsky V., Habler L., Sandbank J., Arenzana-Seisdedos F., Petit I., Ben-Hur H., Lapidot T., Alon R. (1999). The Chemokine SDF-1 Stimulates Integrin-Mediated Arrest of CD34(+) Cells on Vascular Endothelium under Shear Flow. J. Clin. Investig..

[51] Bilgin Y.M. (2021). Use of Plerixafor for Stem Cell Mobilization in the Setting of Autologous and Allogeneic Stem Cell Transplantations: An Update. J. Blood Med..

[52] Dasari A., Shen C., Halperin D., Zhao B., Zhou S., Xu Y., Shih T., Yao J.C. (2017). Trends in the Incidence, Prevalence, and Survival Outcomes in Patients With Neuroendocrine Tumors in the United States. JAMA Oncol..

[53] Das S., Dasari A. (2021). Epidemiology, Incidence, and Prevalence of Neuroendocrine Neoplasms: Are There Global Differences?. Curr. Oncol. Rep..

[54] Modlin I.M., Moss S.F., Chung D.C., Jensen R.T., Snyderwine E. (2008). Priorities for Improving the Management of Gastroenteropancreatic Neuroendocrine Tumors. JNCI J. Natl. Cancer Inst..

[55] Clement D., Ramage J., Srirajaskanthan R. (2020). Update on Pathophysiology, Treatment, and Complications of Carcinoid Syndrome. J. Oncol..

[56] Del Olmo-Garcia M.I., Prado-Wohlwend S., Andres A., Soriano J.M., Bello P., Merino-Torres J.F. (2021). Somatostatin and Somatostatin Receptors: From Signaling to Clinical Applications in Neuroendocrine Neoplasms. Biomedicines.

[57] Gabriel M., Decristoforo C., Kendler D., Dobrozemsky G., Heute D., Uprimny C., Kovacs P., Von Guggenberg E., Bale R., Virgolini I.J. (2007). 68Ga-DOTA-Tyr3-Octreotide PET in Neuroendocrine Tumors: Comparison with Somatostatin Receptor Scintigraphy and CT. J. Nucl. Med..

[58] Rinke A., Müller H.-H., Schade-Brittinger C., Klose K.-J., Barth P., Wied M., Mayer C., Aminossadati B., Pape U.-F., Bläker M. (2009). Placebo-Controlled, Double-Blind, Prospective, Randomized Study on the Effect of Octreotide LAR in the Control of Tumor Growth in Patients with Metastatic Neuroendocrine Midgut Tumors: A Report from the PROMID Study Group. J. Clin. Oncol. Off. J. Am. Soc. Clin. Oncol..

[59] Caplin M.E., Pavel M., Ćwikła J.B., Phan A.T., Raderer M., Sedláčková E., Cadiot G., Wolin E.M., Capdevila J., Wall L. (2014). Lanreotide in Metastatic Enteropancreatic Neuroendocrine Tumors. N. Engl. J. Med..

[60] Baudin E., Horsch D., Singh S., Caplin M.E., Ferone D., Wolin E.M., Capdevila J., Buikhuisen W.A., Raderer M., Dansin E. (2021). 1096O Lanreotide Autogel/Depot (LAN) in Patients with Advanced Bronchopulmonary (BP) Neuroendocrine Tumors (NETs): Results from the Phase III SPINET Study. Ann. Oncol..

[61] Calomino N., Poto G.E., Carbone L., Bagnacci G., Piccioni S., Andreucci E., Nenci L., Marano L., Verre L., Petrioli R. (2023). Neuroendocrine Tumors’ Patients Treated with Somatostatin Analogue Could Complicate with Emergency Cholecystectomy. Ann. Ital. Chir..

[62] Nagtegaal I.D., Odze R.D., Klimstra D., Paradis V., Rugge M., Schirmacher P., Washington K.M., Carneiro F., Cree I.A. (2020). The 2019 WHO Classification of Tumours of the Digestive System. Histopathology.

[63] Travis W.D., Brambilla E., Nicholson A.G., Yatabe Y., Austin J.H.M., Beasley M.B., Chirieac L.R., Dacic S., Duhig E., Flieder D.B. (2015). The 2015 World Health Organization Classification of Lung Tumors: Impact of Genetic, Clinical and Radiologic Advances Since the 2004 Classification. J. Thorac. Oncol. Off. Publ. Int. Assoc. Study Lung Cancer.

[64] Corleto V.D., Falconi M., Panzuto F., Milione M., De Luca O., Perri P., Cannizzaro R., Bordi C., Pederzoli P., Scarpa A. (2009). Somatostatin Receptor Subtypes 2 and 5 Are Associated with Better Survival in Well-Differentiated Endocrine Carcinomas. Neuroendocrinology.

[65] Zamora V., Cabanne A., Salanova R., Bestani C., Domenichini E., Marmissolle F., Giacomi N., O’Connor J., Méndez G., Roca E. (2010). Immunohistochemical Expression of Somatostatin Receptors in Digestive Endocrine Tumours. Dig. Liver Dis. Off. J. Ital. Soc. Gastroenterol. Ital. Assoc. Study Liver.

[66] Okuwaki K., Kida M., Mikami T., Yamauchi H., Imaizumi H., Miyazawa S., Iwai T., Takezawa M., Saegusa M., Watanabe M. (2013). Clinicopathologic Characteristics of Pancreatic Neuroendocrine Tumors and Relation of Somatostatin Receptor Type 2A to Outcomes. Cancer.

[67] Qian Z.R., Li T., Ter-Minassian M., Yang J., Chan J.A., Brais L.K., Masugi Y., Thiaglingam A., Brooks N., Nishihara R. (2016). Association Between Somatostatin Receptor Expression and Clinical Outcomes in Neuroendocrine Tumors. Pancreas.

[68] Mehta S., de Reuver P.R., Gill P., Andrici J., D’Urso L., Mittal A., Pavlakis N., Clarke S., Samra J.S., Gill A.J. (2015). Somatostatin Receptor SSTR-2a Expression Is a Stronger Predictor for Survival Than Ki-67 in Pancreatic Neuroendocrine Tumors. Medicine.

[69] Song K.B., Kim S.C., Kim J.H., Seo D.-W., Hong S.-M., Park K.-M., Hwang D.W., Lee J.H., Lee Y.-J. (2016). Prognostic Value of Somatostatin Receptor Subtypes in Pancreatic Neuroendocrine Tumors. Pancreas.

[70] Circelli L., Sciammarella C., Guadagno E., Tafuto S., Del Basso De Caro M., Botti G., Pezzullo L., Aria M., Ramundo V., Tatangelo F. (2016). CXCR4/CXCL12/CXCR7 Axis Is Functional in Neuroendocrine Tumors and Signals on mTOR. Oncotarget.

[71] Yao J.C., Lombard-Bohas C., Baudin E., Kvols L.K., Rougier P., Ruszniewski P., Hoosen S., St Peter J., Haas T., Lebwohl D. (2010). Daily Oral Everolimus Activity in Patients with Metastatic Pancreatic Neuroendocrine Tumors after Failure of Cytotoxic Chemotherapy: A Phase II Trial. J. Clin. Oncol. Off. J. Am. Soc. Clin. Oncol..

[72] Pavel M.E., Hainsworth J.D., Baudin E., Peeters M., Hörsch D., Winkler R.E., Klimovsky J., Lebwohl D., Jehl V., Wolin E.M. (2011). Everolimus plus Octreotide Long-Acting Repeatable for the Treatment of Advanced Neuroendocrine Tumours Associated with Carcinoid Syndrome (RADIANT-2): A Randomised, Placebo-Controlled, Phase 3 Study. Lancet.

[73] Korbecki J., Simińska D., Gąssowska-Dobrowolska M., Listos J., Gutowska I., Chlubek D., Baranowska-Bosiacka I. (2021). Chronic and Cycling Hypoxia: Drivers of Cancer Chronic Inflammation through HIF-1 and NF-κB Activation: A Review of the Molecular Mechanisms. Int. J. Mol. Sci..

[74] Albadari N., Deng S., Li W. (2019). The Transcriptional Factors HIF-1 and HIF-2 and Their Novel Inhibitors in Cancer Therapy. Expert Opin. Drug Discov..

[75] Wicks E.E., Semenza G.L. (2022). Hypoxia-Inducible Factors: Cancer Progression and Clinical Translation. J. Clin. Investig..

[76] Jin Z., Xiang R., Dai J., Wang Y., Xu Z. (2023). HIF-1α Mediates CXCR4 Transcription to Activate the AKT/mTOR Signaling Pathway and Augment the Viability and Migration of Activated B Cell-like Diffuse Large B-Cell Lymphoma Cells. Mol. Carcinog..

[77] Guo M., Cai C., Zhao G., Qiu X., Zhao H., Ma Q., Tian L., Li X., Hu Y., Liao B. (2014). Hypoxia Promotes Migration and Induces CXCR4 Expression via HIF-1α Activation in Human Osteosarcoma. PLoS ONE.

[78] Arvidsson Y., Bergström A., Arvidsson L., Kristiansson E., Ahlman H., Nilsson O. (2010). Hypoxia Stimulates CXCR4 Signalling in Ileal Carcinoids. Endocr. Relat. Cancer.

[79] Deschamps L., Bacha D., Rebours V., Mebarki M., Bretagnol F., Panis Y., Bedossa P., Ruszniewski P., Couvelard A. (2012). The Expression of the Hypoxia Markers CA9 and CXCR4 Is Correlated with Survival in Patients with Neuroendocrine Tumours of the Ileum. Neuroendocrinology.

[80] Kaemmerer D., Träger T., Hoffmeister M., Sipos B., Hommann M., Sänger J., Schulz S., Lupp A. (2015). Inverse Expression of Somatostatin and CXCR4 Chemokine Receptors in Gastroenteropancreatic Neuroendocrine Neoplasms of Different Malignancy. Oncotarget.

[81] Mai R., Kaemmerer D., Träger T., Neubauer E., Sänger J., Baum R.P., Schulz S., Lupp A. (2019). Different Somatostatin and CXCR4 Chemokine Receptor Expression in Gastroenteropancreatic Neuroendocrine Neoplasms Depending on Their Origin. Sci. Rep..

[82] Popa O., Taban S.M., Dema A.L.C., Plopeanu A.D., Barna R.A., Cornianu M., Dema S. (2021). Immunohistochemical Expression of Chemokine Receptor in Neuroendocrine Neoplasms (CXCR4) of the Gastrointestinal Tract: A Retrospective Study of 71 Cases. Rom. J. Morphol. Embryol..

[83] Kaemmerer D., Reimann C., Specht E., Wirtz R.M., Sayeg M., Baum R.P., Schulz S., Lupp A. (2015). Differential Expression and Prognostic Value of the Chemokine Receptor CXCR4 in Bronchopulmonary Neuroendocrine Neoplasms. Oncotarget.

[84] Lambert A.W., Pattabiraman D.R., Weinberg R.A. (2017). Emerging Biological Principles of Metastasis. Cell.

[85] Galván J.A., Astudillo A., Vallina A., Crespo G., Folgueras M.V., González M.V. (2014). Prognostic and Diagnostic Value of Epithelial to Mesenchymal Transition Markers in Pulmonary Neuroendocrine Tumors. BMC Cancer.

[86] Galván J.A., Astudillo A., Vallina A., Fonseca P.J., Gómez-Izquierdo L., García-Carbonero R., González M.V. (2013). Epithelial-Mesenchymal Transition Markers in the Differential Diagnosis of Gastroenteropancreatic Neuroendocrine Tumors. Am. J. Clin. Pathol..

[87] Fendrich V., Maschuw K., Waldmann J., Buchholz M., Rehm J., Gress T.M., Bartsch D.K., König A. (2012). Epithelial-Mesenchymal Transition Is a Critical Step in Tumorgenesis of Pancreatic Neuroendocrine Tumors. Cancers.

[88] Cives M., Quaresmini D., Rizzo F.M., Felici C., D’Oronzo S., Simone V., Silvestris F. (2017). Osteotropism of Neuroendocrine Tumors: Role of the CXCL12/CXCR4 Pathway in Promoting EMT in Vitro. Oncotarget.

[89] Cives M., Pellè E., Rinzivillo M., Prosperi D., Tucci M., Silvestris F., Panzuto F. (2021). Bone Metastases in Neuroendocrine Tumors: Molecular Pathogenesis and Implications in Clinical Practice. Neuroendocrinology.

[90] Yu J., Li N., Li J., Lu M., Leal J.P., Tan H., Su H., Fan Y., Zhang Y., Zhao W. (2019). The Correlation Between [68Ga]DOTATATE PET/CT and Cell Proliferation in Patients With GEP-NENs. Mol. Imaging Biol..

[91] Ezziddin S., Adler L., Sabet A., Pöppel T.D., Grabellus F., Yüce A., Fischer H.-P., Simon B., Höller T., Biersack H.-J. (2014). Prognostic Stratification of Metastatic Gastroenteropancreatic Neuroendocrine Neoplasms by 18F-FDG PET: Feasibility of a Metabolic Grading System. J. Nucl. Med. Off. Publ. Soc. Nucl. Med..

[92] Abgral R., Leboulleux S., Déandreis D., Aupérin A., Lumbroso J., Dromain C., Duvillard P., Elias D., De Baere T., Guigay J. (2011). Performance of 18Fluorodeoxyglucose-Positron Emission Tomography and Somatostatin Receptor Scintigraphy for High Ki67 (≥10%) Well-Differentiated Endocrine Carcinoma Staging. J. Clin. Endocrinol. Metab..

[93] Pauwels E.K.J., Sturm E.J.C., Bombardieri E., Cleton F.J., Stokkel M.P.M. (2000). Positron-Emission Tomography with [18F]Fluorodeoxyglucose. J. Cancer Res. Clin. Oncol..

[94] Uy G.L., Rettig M.P., Cashen A.F. (2008). Plerixafor, a CXCR4 Antagonist for the Mobilization of Hematopoietic Stem Cells. Expert Opin. Biol. Ther..

[95] Aghanejad A., Jalilian A.R., Fazaeli Y., Alirezapoor B., Pouladi M., Beiki D., Maus S., Khalaj A. (2014). Synthesis and Evaluation of [67Ga]-AMD3100: A Novel Imaging Agent for Targeting the Chemokine Receptor CXCR4. Sci. Pharm..

[96] Gourni E., Demmer O., Schottelius M., D’Alessandria C., Schulz S., Dijkgraaf I., Schumacher U., Schwaiger M., Kessler H., Wester H.-J. (2011). PET of CXCR4 Expression by a 68Ga-Labeled Highly Specific Targeted Contrast Agent. J. Nucl. Med..

[97] Demmer O., Gourni E., Schumacher U., Kessler H., Wester H.-J. (2011). PET Imaging of CXCR4 Receptors in Cancer by a New Optimized Ligand. ChemMedChem.

[98] Martin R., Jüttler S., Müller M., Wester H.-J. (2014). Cationic Eluate Pretreatment for Automated Synthesis of [^68^Ga]CPCR4.2. Nucl. Med. Biol..

[99] Zehnder A., Pickel S., Bouterfa H., Berroteran-Infante N., Buck A. (2022). Diagnostische Leistung und Sicherheit von [68Ga]Ga-Pentixafor zur Erkennung und Lokalisation von Chemokin Rezeptor 4 (CXCR4) positiven Tumoren und Metastasen in einem PAN Cancer Ansatz: Eine prospektive, multizentrische, internationale, klinische Phase III Studie, die FORPAN Studie. Oncol. Res. Treat..

[100] Werner R.A., Weich A., Higuchi T., Schmid J.S., Schirbel A., Lassmann M., Wild V., Rudelius M., Kudlich T., Herrmann K. (2017). Imaging of Chemokine Receptor 4 Expression in Neuroendocrine Tumors—A Triple Tracer Comparative Approach. Theranostics.

[101] Werner R.A., Weich A., Schirbel A., Samnick S., Buck A.K., Higuchi T., Wester H.-J., Lapa C. (2017). Intraindividual Tumor Heterogeneity in NET—Further Insight by C-X-C Motif Chemokine Receptor 4-Directed Imaging. Eur. J. Nucl. Med. Mol. Imaging.

[102] Weich A., Werner R.A., Buck A.K., Hartrampf P.E., Serfling S.E., Scheurlen M., Wester H.-J., Meining A., Kircher S., Higuchi T. (2021). CXCR4-Directed PET/CT in Patients with Newly Diagnosed Neuroendocrine Carcinomas. Diagnostics.

[103] Watts A., Singh B., Singh H., Bal A., Kaur H., Dhanota N., Arora S.K., Mittal B.R., Behera D. (2023). [68Ga]Ga-Pentixafor PET/CT Imaging for in Vivo CXCR4 Receptor Mapping in Different Lung Cancer Histologic Sub-Types: Correlation with Quantitative Receptors’ Density by Immunochemistry Techniques. Eur. J. Nucl. Med. Mol. Imaging.

[104] Lewis R., Habringer S., Kircher M., Hefter M., Peuker C.A., Werner R., Ademaj-Kospiri V., Gäble A., Weber W., Wester H.-J. (2021). Investigation of Spleen CXCR4 Expression by [68Ga]Pentixafor PET in a Cohort of 145 Solid Cancer Patients. EJNMMI Res..

[105] Chatterjee S., Behnam Azad B., Nimmagadda S. (2014). The Intricate Role of CXCR4 in Cancer. Advances in Cancer Research.

[106] Wang Z., Ma Q. (2007). Beta-Catenin Is a Promising Key Factor in the SDF-1/CXCR4 Axis on Metastasis of Pancreatic Cancer. Med. Hypotheses.

[107] Kim J.T., Li J., Jang E.R., Gulhati P., Rychahou P.G., Napier D.L., Wang C., Weiss H.L., Lee E.Y., Anthony L. (2013). Deregulation of Wnt/β-Catenin Signaling through Genetic or Epigenetic Alterations in Human Neuroendocrine Tumors. Carcinogenesis.

[108] Weich A., Rogoll D., Gawlas S., Mayer L., Weich W., Pongracz J., Kudlich T., Meining A., Scheurlen M. (2021). Wnt/β-Catenin Signaling Regulates CXCR4 Expression and [68Ga] Pentixafor Internalization in Neuroendocrine Tumor Cells. Diagnostics.

[109] Martin M., Mayer I.A., Walenkamp A.M.E., Lapa C., Andreeff M., Bobirca A. (2021). At the Bedside: Profiling and Treating Patients with CXCR4-Expressing Cancers. J. Leukoc. Biol..

[110] Otani Y., Kijima T., Kohmo S., Oishi S., Minami T., Nagatomo I., Takahashi R., Hirata H., Suzuki M., Inoue K. (2012). Suppression of Metastases of Small Cell Lung Cancer Cells in Mice by a Peptidic CXCR4 Inhibitor TF14016. FEBS Lett..

[111] Salgia R., Stille J.R., Weaver R.W., McCleod M., Hamid O., Polzer J., Roberson S., Flynt A., Spigel D.R. (2017). A Randomized Phase II Study of LY2510924 and Carboplatin/Etoposide versus Carboplatin/Etoposide in Extensive-Disease Small Cell Lung Cancer. Lung Cancer Amst. Neth..

[112] Robinson T., Escara-Wilke J., Dai J., Zimmermann J., Keller E.T. (2023). A CXCR4 Inhibitor (Balixafortide) Enhances Docetaxel-Mediated Antitumor Activity in a Murine Model of Prostate Cancer Bone Metastasis. Prostate.

[113] Bockorny B., Macarulla T., Semenisty V., Borazanci E., Feliu J., Ponz-Sarvise M., Abad D.G., Oberstein P., Alistar A., Muñoz A. (2021). Motixafortide and Pembrolizumab Combined to Nanoliposomal Irinotecan, Fluorouracil, and Folinic Acid in Metastatic Pancreatic Cancer: The COMBAT/KEYNOTE-202 Trial. Clin. Cancer Res. Off. J. Am. Assoc. Cancer Res..

[114] Andtbacka R.H.I., Wang Y., Pierce R.H., Campbell J.S., Yushak M., Milhem M., Ross M., Niland K., Arbeit R.D., Parasuraman S. (2022). Mavorixafor, an Orally Bioavailable CXCR4 Antagonist, Increases Immune Cell Infiltration and Inflammatory Status of Tumor Microenvironment in Patients with Melanoma. Cancer Res. Commun..

[115] Karpova D., Bräuninger S., Wiercinska E., Krämer A., Stock B., Graff J., Martin H., Wach A., Escot C., Douglas G. (2017). Mobilization of Hematopoietic Stem Cells with the Novel CXCR4 Antagonist POL6326 (Balixafortide) in Healthy Volunteers-Results of a Dose Escalation Trial. J. Transl. Med..

[116] Crees Z.D., Rettig M.P., Jayasinghe R.G., Stockerl-Goldstein K., Larson S.M., Arpad I., Milone G.A., Martino M., Stiff P., Sborov D. (2023). Motixafortide and G-CSF to Mobilize Hematopoietic Stem Cells for Autologous Transplantation in Multiple Myeloma: A Randomized Phase 3 Trial. Nat. Med..

[117] Badolato R., Donadieu J. (2023). Results of a Phase 3 Trial of an Oral CXCR4 Antagonist, Mavorixafor, for Treatment of Patients With WHIM Syndrome. Clin. Immunol..

[118] Bobkov V., Arimont M., Zarca A., De Groof T.W.M., Van Der Woning B., De Haard H., Smit M.J. (2019). Antibodies Targeting Chemokine Receptors CXCR4 and ACKR3. Mol. Pharmacol..

[119] Pellé E., Cives M., Quaresmini D., Lovero D., Felici C., Cafforio P., Palmirotta R., Silvestris F. (2017). CXCR4 Inhibition by Ulocuplumab Prevents EMT of pNET Cells in Vitro. Ann. Oncol..

[120] Si Y., Guan J., Xu Y., Chen K., Kim S., Zhou L., Jaskula-Sztul R., Liu X.M. (2020). Dual-Targeted Extracellular Vesicles to Facilitate Combined Therapies for Neuroendocrine Cancer Treatment. Pharmaceutics.

[121] Sabet A., Mader N., Bittenbring J.T., Khreish F., Grünwald F., Biersack H.J., Ezziddin S. (2021). Prophylactic Peripheral Blood Stem Cell Collection in Patients with Extensive Bone-Marrow Infiltration of Neuroendocrine Tumours Prior to Peptide Receptor Radionuclide Therapy with 177Lu-DOTATATE. Pharmaceuticals.

[122] Strosberg J., El-Haddad G., Wolin E., Hendifar A., Yao J., Chasen B., Mittra E., Kunz P.L., Kulke M.H., Jacene H. (2017). Phase 3 Trial of 177Lu-Dotatate for Midgut Neuroendocrine Tumors. N. Engl. J. Med..

[123] Strosberg J.R., Caplin M.E., Kunz P.L., Ruszniewski P.B., Bodei L., Hendifar A., Mittra E., Wolin E.M., Yao J.C., Pavel M.E. (2021). 177Lu-Dotatate plus Long-Acting Octreotide versus High-dose Long-Acting Octreotide in Patients with Midgut Neuroendocrine Tumours (NETTER-1): Final Overall Survival and Long-Term Safety Results from an Open-Label, Randomised, Controlled, Phase 3 Trial. Lancet Oncol..

[124] Poschenrieder A., Schottelius M., Schwaiger M., Kessler H., Wester H.-J. (2016). The Influence of Different Metal-Chelate Conjugates of Pentixafor on the CXCR4 Affinity. EJNMMI Res..

[125] Schottelius M., Osl T., Poschenrieder A., Hoffmann F., Beykan S., Hänscheid H., Schirbel A., Buck A.K., Kropf S., Schwaiger M. (2017). [177Lu]Pentixather: Comprehensive Preclinical Characterization of a First CXCR4-Directed Endoradiotherapeutic Agent. Theranostics.

[126] Herrmann K., Schottelius M., Lapa C., Osl T., Poschenrieder A., Hänscheid H., Lückerath K., Schreder M., Bluemel C., Knott M. (2016). First-in-Human Experience of CXCR4-Directed Endoradiotherapy with 177Lu- and 90Y-Labeled Pentixather in Advanced-Stage Multiple Myeloma with Extensive Intra- and Extramedullary Disease. J. Nucl. Med..

[127] Lapa C., Kircher S., Schirbel A., Rosenwald A., Kropf S., Pelzer T., Walles T., Buck A.K., Weber W.A., Wester H.-J. (2017). Targeting CXCR4 with [68Ga]Pentixafor: A Suitable Theranostic Approach in Pleural Mesothelioma?. Oncotarget.

[128] Lapa C., Hänscheid H., Kircher M., Schirbel A., Wunderlich G., Werner R.A., Samnick S., Kotzerke J., Einsele H., Buck A.K. (2019). Feasibility of CXCR4-Directed Radioligand Therapy in Advanced Diffuse Large B-Cell Lymphoma. J. Nucl. Med. Off. Publ. Soc. Nucl. Med..

[129] Habringer S., Lapa C., Herhaus P., Schottelius M., Istvanffy R., Steiger K., Slotta-Huspenina J., Schirbel A., Hänscheid H., Kircher S. (2018). Dual Targeting of Acute Leukemia and Supporting Niche by CXCR4-Directed Theranostics. Theranostics.

[130] Jacobs S.M., Wesseling P., de Keizer B., Tolboom N., Ververs F.F.T., Krijger G.C., Westerman B.A., Snijders T.J., Robe P.A., van der Kolk A.G. (2022). CXCR4 Expression in Glioblastoma Tissue and the Potential for PET Imaging and Treatment with [68Ga]Ga-Pentixafor/[177Lu]Lu-Pentixather. Eur. J. Nucl. Med. Mol. Imaging.

[131] Fath M.A., Liu D., Ewald J.T., Robles-Planells C., Tomanek-Chalkley A.M., Graves S.A., Howe J.R., O’Dorisio T.M., Rastogi P., Bellizzi A.M. (2023). Chemokine Receptor CXCR4 Radioligand Targeted Therapy Using 177Lutetium-Pentixather for Pulmonary Neuroendocrine Cancers. Radiat. Res..

[132] Fath M.A., Liu D., Robles-Planells C., Ewald J.T., Christensen K.A., Johnson S.S., Graves S.A., Spitz D.R., Menda Y., Sue O’Dorisio M. (2023). Abstract 5034: Targeting CXCR4 and Thioredoxin Reductase in High Grade Neuroendocrine Tumors and Neuroendocrine Carcinomas. Cancer Res..

